# Primary Invasive Squamous Cell Carcinoma of the Nipple

**DOI:** 10.1155/2015/327487

**Published:** 2015-12-21

**Authors:** Avani A. Pendse, Siobhan M. O'Connor

**Affiliations:** Department of Pathology, University of North Carolina, Chapel Hill, NC 27599-7525, USA

## Abstract

Squamous cell carcinoma is one of the most common cutaneous cancers; however, primary squamous cell carcinoma of the nipple is extremely rare. Among the few reported cases, the majority have occurred in older women with rare cases seen in younger women and male patients. Our patient presented with an exophytic mass of the right nipple while pregnant. A superficial biopsy was reviewed at an outside institution and then at our institution and diagnosed as squamous papilloma and then as hyperkeratosis of the nipple, respectively. The subsequent excisional biopsy revealed multiple nests of tumor cells extending into the dermis with associated chronic inflammatory infiltrate, and the lesion was diagnosed as a primary invasive squamous cell carcinoma of the nipple. Following that, a wide local excision of the excision site and sampling of the regional lymph nodes were negative for carcinoma. Due to the rarity of this diagnosis, it is not known whether prognosis and response to therapy differ from cutaneous squamous cell carcinoma at other sites. Therefore, risk stratification and therapy have been based on those for cutaneous squamous cell carcinoma.

## 1. Introduction

Primary squamous cell carcinoma of the nipple is an extremely rare condition, with only nine cases reported in the English literature [1–8]. The majority of the previously reported cases have been in older women with an age range of 62 to 87 years with rare occurrences in younger individuals. Of the younger patients, one was male and positive for human immunodeficiency virus (HIV) [7] and the other was female with no known risk factors [1]. Here, we report a well-differentiated primary invasive squamous cell carcinoma of the nipple occurring in a young pregnant woman.

## 2. Case Presentation

**Figure 1:**
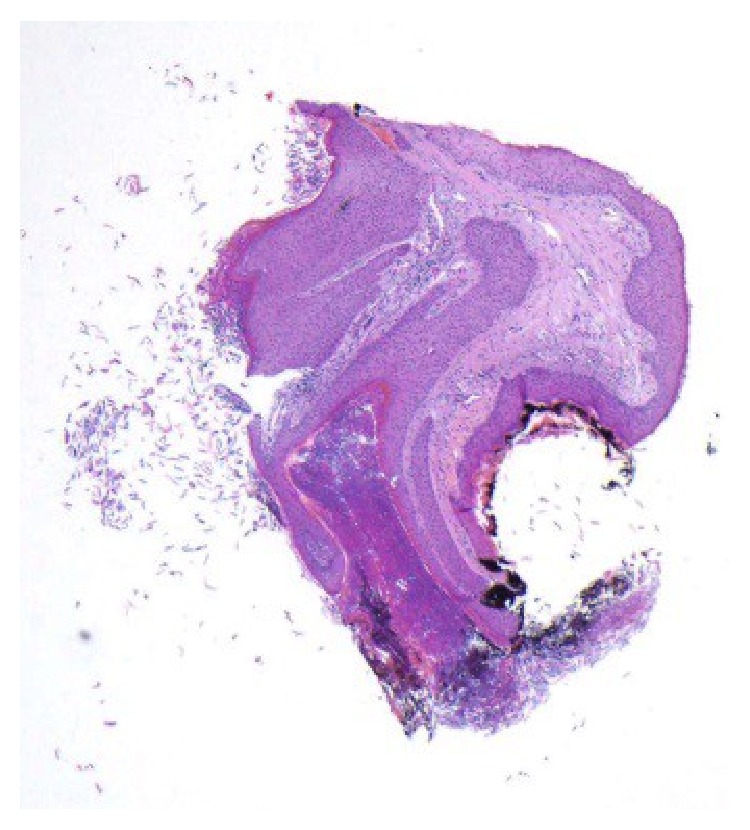
Low power view of the initial biopsy (HE, 4x).

**Figure 2:**
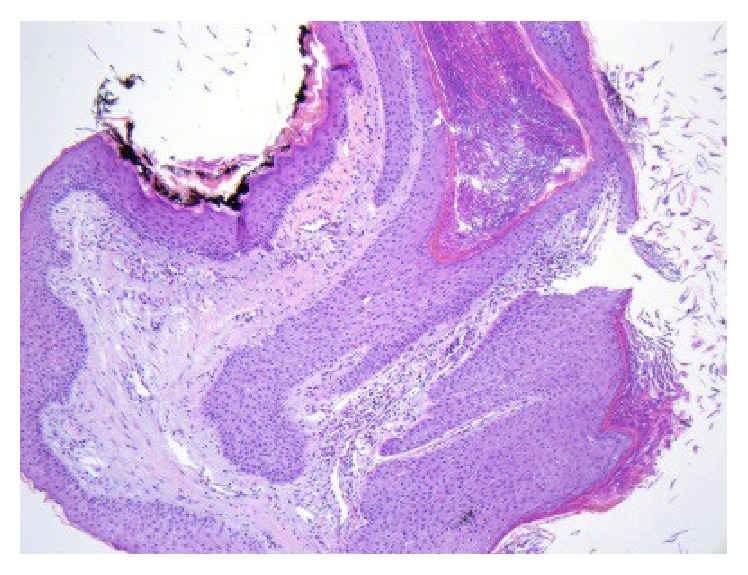
Initial biopsy showing acanthosis, hyperkeratosis, and keratotic plugging (HE, 10x).

The patient is a 29-year-old pregnant female, who originally presented with a small nodule on the tip of her right nipple, at approximately 28 weeks of gestation. The lesion increased in size over the next few weeks and showed no improvement with a 2-week course of antibiotic treatment (Keflex). Due to the progressive increase in the size of the lesion, the patient was referred to a general surgeon and underwent an incisional biopsy. As seen in Figures 1 and 2, the biopsy showed fragments of squamous epithelium with hyperkeratosis, keratotic plugging, and filiform acanthosis. These features were interpreted as consistent with a squamous papilloma. On review at our institution, we agreed with the originating pathologist's description of the lesion. However, due to the patient's clinical history and the degree of hyperkeratosis and keratotic plugging, in our opinion, findings were more consistent with hyperkeratosis of the nipple.

**Figure 3:**
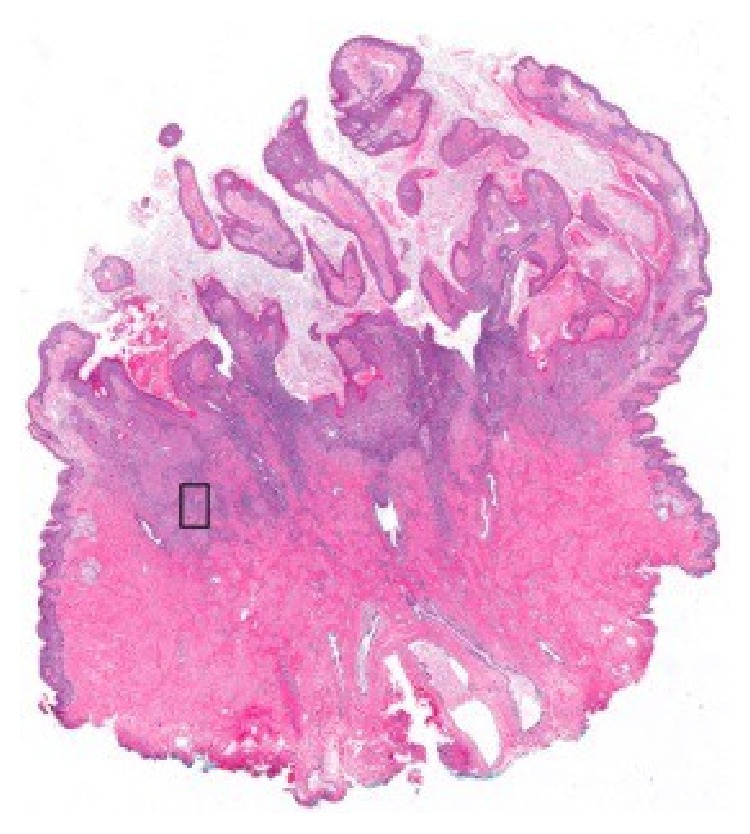
Whole slide image of the squamous cell carcinoma showing the keratoacanthoma-like features (HE). The box indicates the area shown in Figure 4.

**Figure 4:**
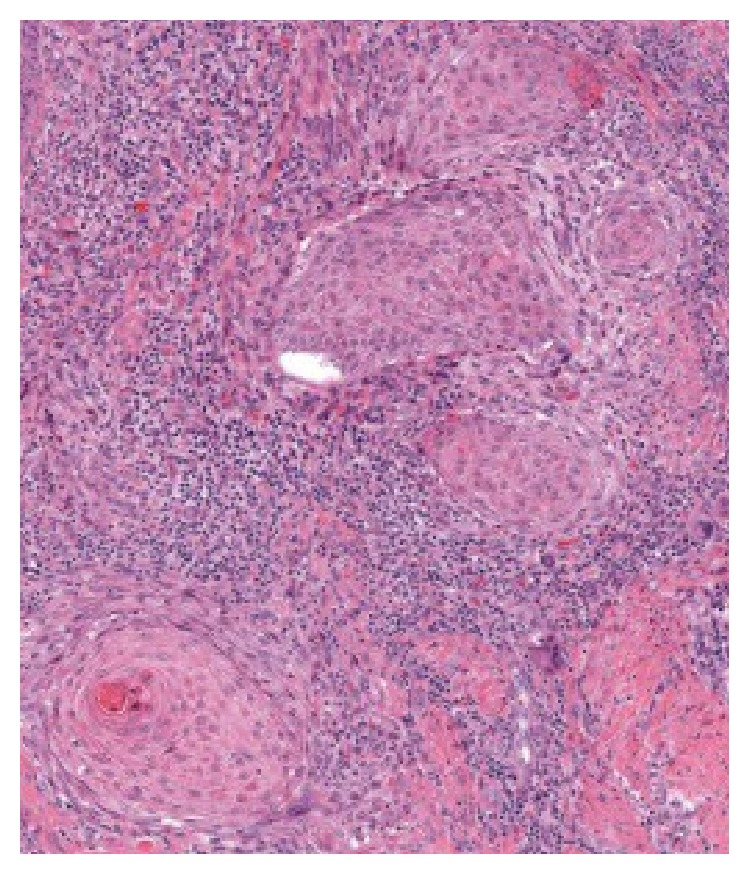
Area extracted from the whole slide image showing nests of invasion and surrounding inflammation (HE).

The patient delivered a healthy infant but was unable to nurse on the right side due to sloughing of the nipple skin and pain. In addition, the lesion continued to increase in size. On physical examination at our institution, a soft, exophytic mass of the right nipple was noted. The areola was uninvolved. No other abnormalities in either breast and no axillary lymphadenopathy were detected. A mammogram was performed and revealed no masses, calcifications, or sites of architectural distortion within the breast parenchyma. The radiologist noted that, due to the heterogeneously dense breast composition, small masses could be obscured and a targeted ultrasound was performed. The ultrasound revealed an enlarged right nipple, approximately 1.7 cm in diameter with a hypervascular nipple mass. The subareolar ducts were normal in size, without evidence of an intraductal mass. Although the previous sample had been interpreted as benign, the biopsy was superficial, and, given the progressive increase in size, excision of the lesion was recommended. The patient underwent excisional biopsy of the right nipple lesion and, on gross examination, the nipple showed an ill-defined soft yellow/white friable verrucous mass measuring 2.4 × 1.6 × 1.1 cm. Histology revealed an exuberant squamous proliferation with exophytic growth pattern, marked hyperkeratosis, acanthosis, and collarettes at the periphery, all features suggestive of a keratoacanthoma (Figure 3). Multiple irregular nests of tumor extended into the dermis along with rare infiltrating single cells (Figure 4). Maximum tumor thickness was 1.4 cm with depth of invasion 4 mm.

**Figure 5:**
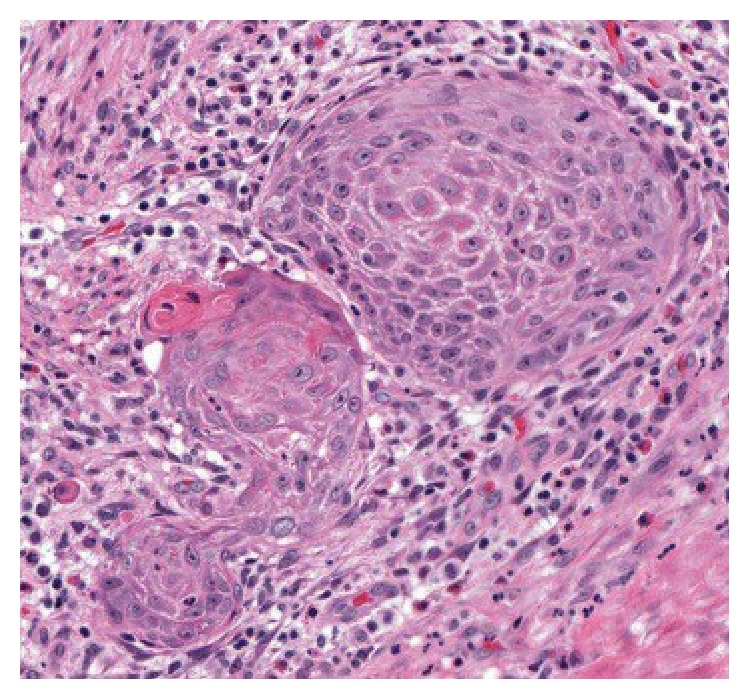
Area extracted from the whole slide image showing cytologic detail and mitotic figure (HE).

**Figure 6:**
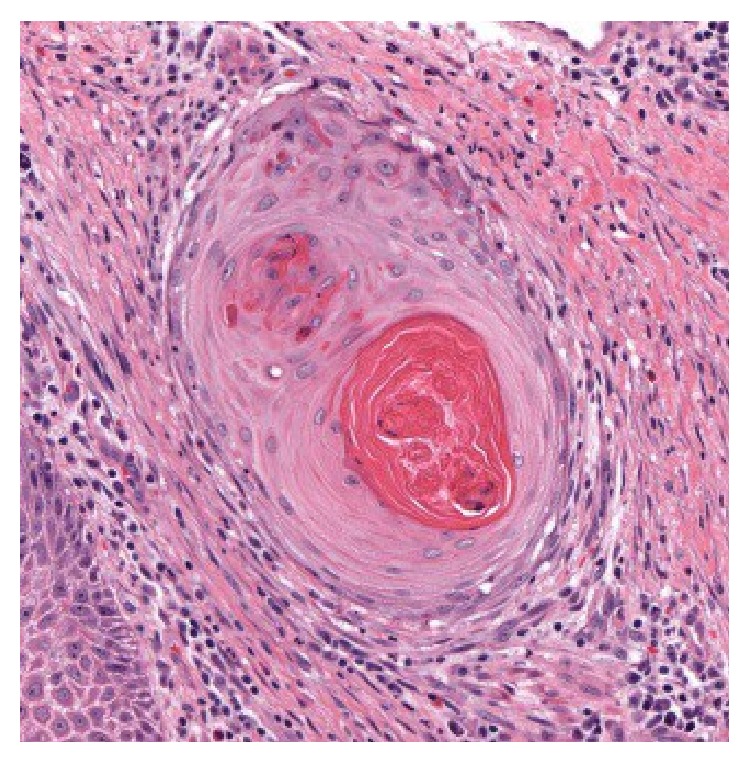
Area extracted from the whole slide image showing desmoplastic response (HE).

**Figure 7:**
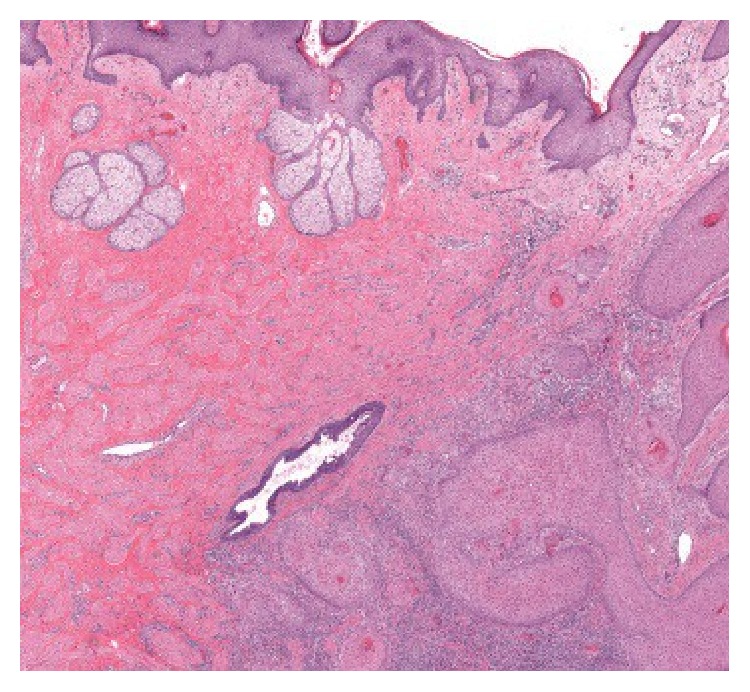
Area extracted from the whole slide image showing extension of tumor deep to the sebaceous glands and a lactiferous duct (HE).

**Figure 8:**
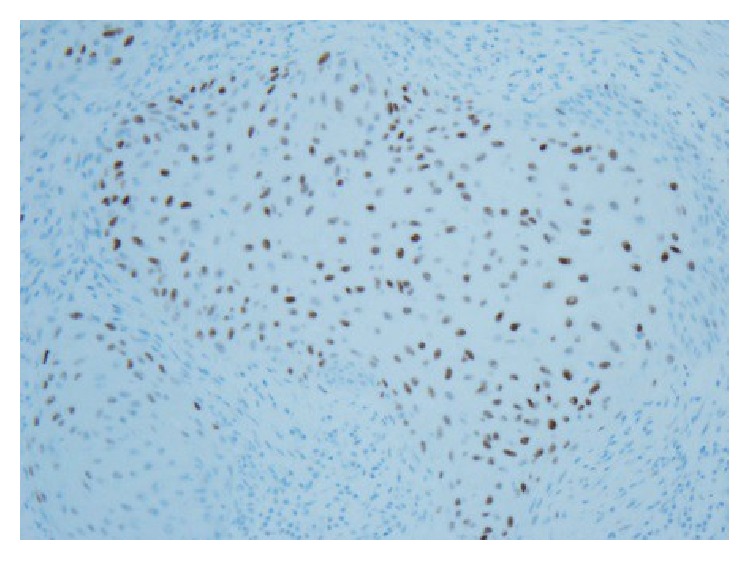
Positive nuclear immunostaining for p53 in invasive nests (p53, 20x).

The distinction between keratoacanthoma and squamous cell carcinoma remains difficult and controversial. Features of our lesion that favor invasive squamous cell carcinoma include prominent nuclear atypia, mitotic figures (Figure 5), and desmoplastic stromal response [9] (Figure 6). In addition, keratoacanthoma does not typically extend beyond the level of the sweat glands [9]. There are no sweat glands present in our specimen, but Figure 7 shows extension of the tumor deep to the sebaceous glands and a lactiferous duct. The infiltrating nests were positive for p16 immunohistochemical stain, but in situ hybridization for high risk HPV was negative. Immunostain for p53 was diffusely positive in the infiltrating nests (Figure 8). One study showed a significant trend in the proportion of p53 reactivity from keratoacanthomas to poorly differentiated squamous cell carcinomas, although p53 reactivity could not reliably distinguish between keratoacanthoma and well-differentiated squamous cell carcinoma [10]. Another series showed more frequent p53 expression in squamous cell carcinomas with keratoacanthoma like features versus well-differentiated squamous cell carcinomas and keratoacanthomas, but the differences did not reach statistical significance [11]. Taken all together, features supported the diagnosis of squamous cell carcinoma with a keratoacanthoma-like growth pattern rather than a keratoacanthoma.

Subsequently, the patient underwent a wide local excision with regional lymph node dissection. The entire excision specimen was submitted for histopathologic evaluation and was negative for carcinoma. Regional lymph nodes were also negative for metastatic disease. The patient has no evidence of recurrence after 15 months.

## 3. Discussion

Squamous cell carcinoma is one of the most common cutaneous cancers and causes significant morbidity, particularly in elderly patients [12]. Exposure to ultraviolet radiation and lighter skin color are well-established risk factors for cutaneous squamous cell carcinoma, along with an emerging role for human papillomavirus infection [13]. Primary squamous cell carcinoma of the nipple is rare, possibly due to less exposure of this area to sunlight.

The previous case reports are summarized in Table 1. Of the nine reported cases, three were in situ [4, 7, 8] and one showed <1 mm of invasion [5]. Six carcinomas (in situ or invasive) occurred in postmenopausal women ranging in age from 62 to 87 years [3–6, 8] and one invasive carcinoma was found in a 62-year-old male [2]. Of the two cases reported in younger patients, one was in a 41-year-old male who was HIV positive (in situ) [7] and one was in a 34-year-old female (invasive) [1]. Given the rarity of primary squamous cell carcinoma of the nipple and areola, firm causative or correlative relationships with genetic and environmental risk factors have not been established. Similarly, the natural history of this disease is largely unknown. Our patient has a history of hormonal contraceptive use, although no exposure to other hormones was reported. Family history was also negative for skin and breast malignancy. Known risk factors were identified in four of the nine previous patients, a male with HIV [7], a male with work associated sun exposure over a 10-year period [2], and two women who had previously undergone radiation therapy for breast carcinoma [3, 8].

In the previously reported cases of squamous cell carcinoma of the nipple, two patients presented with an exophytic mass [5, 6]. In the remaining patients, physical examination revealed scaling, erythema, and/or ulceration, which raised the possibility of Paget disease of the nipple, a much more common entity [1–4, 7, 8]. The exophytic nature of the lesion in our patient made Paget disease less likely but instead was suggestive of hyperkeratotic processes such as nevoid hyperkeratosis and, particularly in our patient, pregnancy associated hyperkeratosis. In fact, our patient's initial biopsy was interpreted as changes consistent with hyperkeratosis but was not subcategorized since clinical presentation and histology did not fit neatly into a specific subtype.

Nevoid hyperkeratosis is a rare lesion and, in one small series [14], presented as a pigmented hyperkeratotic plaque diffusely involving the nipple and/or areola. Typical findings on histologic examination include orthokeratotic hyperkeratosis, filiform acanthosis, papillomatosis, and occasional keratotic plugging. It is a benign lesion most commonly occurring in women in the second or third decade, essentially asymptomatic, and tends to persist if not treated. Two patients in this series showed improvement with topical retinoic acid but recurred when the treatment was discontinued [14].

In the context of our case, pregnancy associated hyperkeratosis was a consideration based on the initial biopsy. In one case series of pregnancy associated hyperkeratosis, most of the lesions were yellow to tan or mildly pigmented, hyperkeratotic, and/or warty papules on clinical examination [15]. All patients had bilateral involvement, and most lesions were located at the top of the nipple. In contrast to nevoid hyperkeratosis, the majority of patients were symptomatic with complaints including mild tenderness, sensitivity to touch, or mild pruritus. Lesions persisted in the postpartum period in the majority of patients. Histologically, orthokeratotic hyperkeratosis was the most typical feature of pregnancy associated hyperkeratosis with papillomatosis and acanthosis being mild to absent [15].

**Figure 9:**
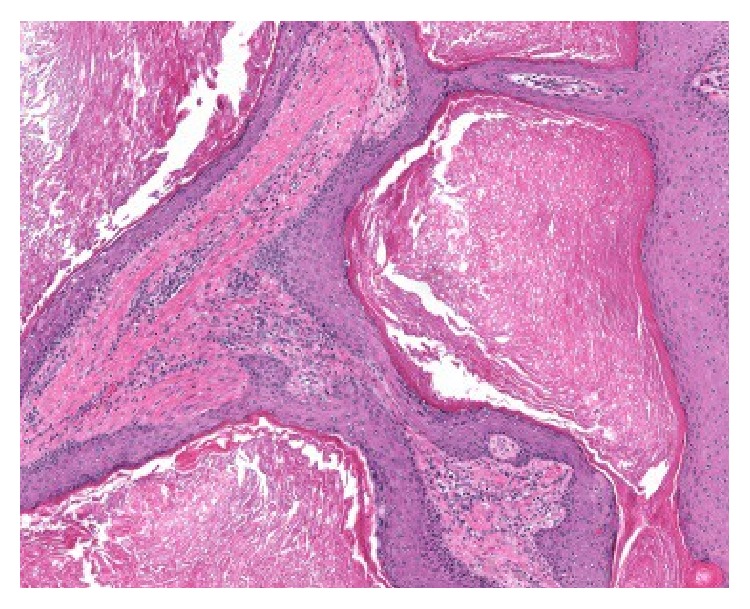
Superficial aspect of resected specimen (HE, 20x).

In general, invasive squamous cell carcinoma of the nipple is easily distinguishable from hyperkeratosis of the nipple. In this case, the initial diagnosis of a hyperkeratotic rather than invasive entity was due to the superficial nature of the biopsy. As is evident in comparing Figures 2 and 9, the features seen in the initial biopsy (Figure 2) are identical to those seen in the superficial aspect of the resected lesion (Figure 9). Once the entire lesion was available for review, it became clear that it was invasive, and benign hyperkeratotic lesions were no longer included in the differential diagnosis. The contrast between the initial biopsy and the subsequent resection specimen dramatically emphasizes the impact that sampling may have on diagnostic accuracy.

In our patient, histologic examination failed to reveal lymphovascular or perineural invasion and the carcinoma was staged based on the American Joint Committee on Cancer (AJCC) staging for cutaneous squamous cell carcinoma [16]. However, it is unknown whether these staging criteria have the same prognostic value in this unique location. Likewise, it is unclear whether standard treatment recommendations are applicable to squamous cell carcinoma of the nipple. Depending on the risk category, the primary treatment options included in the National Comprehensive Cancer Network (NCCN) Guidelines include curettage and electrodissection, surgical excision with 4 to 6 mm margins, or excision with wider surgical margins [17].

Eight of the previously reported patients with squamous cell carcinoma were treated with surgery [1–8], either wide local excision or mastectomy and one patient with in situ disease underwent photodynamic therapy with 5-aminolevulinic acid as a photosensitizer [8]. The latter patient had small areas of residual carcinoma, which were treated with cryotherapy, and she was recurrence-free at 9 months. Of the five patients who underwent wide local excision, one recurred three months postoperatively. The patient underwent repeat wide excision and was free of recurrence at 6 months [8]. Two patients who were treated with mastectomy were free of tumor at 3 and 5 years, respectively [5, 7].

## 4. Conclusion

Primary squamous cell carcinoma of the nipple is a very rare diagnosis. Occurrence of this lesion in a young pregnant woman with no known risk factors for cutaneous cancer made this case all the more challenging. This example highlights the importance of considering rare entities in the differential diagnosis of a hyperkeratotic lesion of the nipple. We hope that our report of a primary squamous cell carcinoma of the nipple in a young pregnant woman will help further understanding of this rare entity.

## Figures and Tables

**Table 1:** Case reports, squamous cell carcinoma of the nipple and areola.

	Patient	Risk factors	Carcinoma	Therapy	Outcome
Venkataseshan et al. [4]	84 y.o. F	Not known	In situ	WLE	DF × 30 months

Sharma and Iyer [7]	41 y.o. M	HIV disease	In situ Negative LNs	Mastectomy, SLN	DF × 3 years

Brookes et al. [8]	71 y.o. F	XRT	In situ	Photodynamic	Residual × 2, cryotherapy DF × 9 months
	69 y.o. F	Not known	Invasive	WLE	Recurrence at 3 months, repeat excision DF × 6 months

Loveland-Jones et al. [3]	66 y.o. F	XRT	Invasive	WLE	Not reported

Hosaka et al. [5]	73 y.o. F	Not known	Superficially invasive (<1 mm) Negative LNs	Mastectomy, ALND	DF × 5 years

Upasham et al. [6]	87 y.o. F	Not known	Invasive Positive LN 1/11	Mod. rad. mastectomy	Not reported

Sofos et al. [1]	34 y.o. F	Not known	Invasive	WLE	DF × 12 months

King and Kremer [2]	62 y.o. M	Sun exposure × 10 years	Invasive with NE differentiation	WLE	Not reported

XRT = radiation therapy; LN = lymph node; SLN = sentinel lymph node; ALND = axillary lymph node dissection; WLE = wide local excision; DF = disease-free.
